# Patients in general practice share a common pattern of symptoms that is partly independent of the diagnosis

**DOI:** 10.1080/02813432.2021.1913886

**Published:** 2021-04-27

**Authors:** Mona Kjeldsberg, Hedda Tschudi-Madsen, Ibrahimu Mdala, Dag Bruusgaard, Bård Natvig

**Affiliations:** General Practice Research Unit (AFE), Department of General Practice, Institute of Health and Society, University of Oslo, Oslo, Norway

**Keywords:** Self-report, symptom reporting, social security grant, questionnaire, general practice

## Abstract

**Objective:**

To describe self-reported symptoms among patients in general practice and to explore the relationships between symptoms experienced by patients and diagnoses given by general practitioners.

**Design:**

Doctor–patient questionnaires focusing on patients’ self-reported symptoms during the past 7 days and the doctors’ diagnoses.

**Setting:**

General practices in urban and suburban areas in Southeast Norway.

**Subjects:**

Forty-seven general practitioners who included 866 patients aged ≥18 years on a random day in practice.

**Results:**

The most frequently reported symptoms were tiredness (46%), lower back pain (43%), neck pain (41%), headache (39%), shoulder pain (36%), and sleep problems (35%). Women had a significantly higher prevalence than men for 16 of 38 symptoms (*p* < 0.05). The mean number of symptoms was 7.5 (range, 0–32; women, 8.1; men, 6.5, *p* < 0.05). Regression analysis showed that patients who received a social security grant had 59% more symptoms than those who were employed and that people with asthenia and depression/anxiety had 44% and 23% more symptoms, respectively than those with all other diagnoses. The patterns of symptoms reported showed similar patterns across the five most prevalent diagnoses.

**Conclusions:**

Patients in general practice report a number of symptoms and share a common pattern of symptoms, which appear to be partly independent of the diagnoses given. These findings suggest that symptoms are not necessarily an indication of disease.

**KEY POINTS**
- Patients consulting general practitioners have a high number of self-reported symptoms.
- The most frequent symptoms are tiredness, lower back pain, neck pain, headache, shoulder pain, and sleep problems.
- Patients diagnosed with asthenia and depression/anxiety report the highest number of symptoms.
- Selected diagnoses show similar patterns in symptom distribution.
- Symptoms are not necessarily an indication of disease.

## Introduction

The presentation of symptoms is the patient’s first step in the communication with the general practitioner (GP) in the consultation. However, the health-care-seeking behaviour of patients does not necessarily reflect the severity of their symptoms. There is a known discrepancy between the full range of symptoms experienced by patients and the symptoms they choose to present to their doctor [1].

How a symptom is interpreted by a person depends on individual factors and preconceptions. The intensity and duration of the symptoms, and the person’s evaluation of the seriousness of the symptoms are central factors in the decision to consult a medical practitioner [2]. The factors that ultimately trigger consultation with a GP vary greatly between patients.

GPs can only evaluate concerns or symptoms presented by the patient. Only 5–25% of people contact a GP because of a symptom [1]. This implies that only a selection of the symptoms is deemed alarming or bothersome enough to motivate the patient to consult a doctor [3]. Even among people with symptoms that could indicate a serious disease, such as blood in the urine, shortness of breath, or coughing blood, only half contact their GP about that symptom [4]. On the other hand, GPs tend to overestimate to what extent their patients consult them for minor medical problems [5].

There is a known discrepancy between the full range of symptoms experienced by patients and the symptoms they choose to present to the doctor [1]. Among patients who disclose, before the consultation, that they have symptoms they intend to discuss with their GP, 23% of the symptoms [6] and up to 25% of patient concerns are not mentioned during the consultation [7]. By contrast, people who consider themselves healthy may report a higher number of symptoms [8]. In most patients who undertake a routine check-up in general practice and who initially declare that they did not have any symptoms, symptoms were found to constitute a major part of their agenda [9].

The commonly used definitions of symptoms relate symptoms to health problems or disease [10]. The WONCA Dictionary of General/Family Practice describes symptoms as, ‘any subjective evidence of a health problem as perceived by the patient’ [11]. Labelling a health problem can result in a disease diagnosis, such as diabetes, or may result in a symptom diagnosis, such as lower back pain or fatigue, both of which group patients under a label describing the main symptom according to the International Classification of Primary Care (ICPC) [12].

Understanding of the process from first experiencing a symptom to receiving a diagnosis is limited. Disentangling the various roles of symptoms in making a diagnosis represents a challenge, and symptoms selected by the patient for presentation to the GP may or may not provide clues relevant to making the diagnosis. Whether a presented symptom will contribute to the final diagnosis depends on whether the GP finds the symptom relevant during the consultation. Although the diagnoses given by GPs have been found to correspond well with their notes about the patient recorded during consultations [13], the diagnoses reflect only the symptoms that are communicated. Other symptoms that are not communicated, commonly referred to as the ‘submerged’ part of a symptom ‘iceberg’, may not be considered [14,15].

More than 20 years ago, Kroenke noted that more diagnostic information may be collected from a patient’s symptom account than from the physical examination [16]. Since then, an increasing number of studies of symptoms have been conducted, but these have focused mainly on symptom prevalence. More recently, it has been acknowledged that both the type and number of symptoms can provide important information about the patient [17]. Counting symptoms, an approach that has been used to study the relationship between functional ability and health in the population, has shown that reporting a high number of symptoms is associated with an increased risk of reporting poor health [18] and may predict future disability benefits [19], irrespective of the type and severity of the symptoms [18,19]. It has also been shown that the number of symptoms may provide more information about future health outcomes than the diagnosis given [20]. These findings suggest that counting symptoms may be a valuable tool in general practice.

We conducted a survey among GPs and their patients in which we focused on symptoms, diagnoses, and function. Our aims were to map the occurrence of a range of common symptoms in patients, regardless of the reasons for the consultation, and to explore the associations between the patient-reported symptoms and the GP-recorded main diagnosis.

## Method

We recruited GPs from meetings with counselling groups for doctors seeking to become specialists in general practice in urban and suburban areas in Southeast Norway. The inclusion period was from June 2010 to January 2012. After a brief introduction to our study, 47 GPs agreed to participate. The GPs were asked to include all consecutive patients regardless of their reason for the encounter, adding up to ≥20 patients aged ≥18 years seen on a random day of practice during the following 2 weeks. If the GP saw <20 patients on a practice day, the inclusion should continue the next practice day. At the end of each consultation, the GPs asked their patients to complete a questionnaire. The patients orally consented to participate after reading the letter accompanying the survey.

The questionnaires for both the GPs and the patients were completed separately directly after the consultation. The answers were linked by serial numbers. A pilot study was first conducted to validate and adjust the questionnaires.

### Dependent variables

The patients were asked whether during the previous week they had experienced any of 38 common symptoms included in a symptom check-list without regard to whether the symptoms were discussed with the GP. The list of symptoms comprised 10 pain symptoms from the Standardised Nordic Questionnaire [21] and 28 symptoms from the Subjective Health Complaints Inventory [22] (Table 1). The dependent variables were the sum score of symptoms and the individual symptoms.

**Table 1.** Distribution of the 38 symptoms reported in general practice for the past 7 days for the total sample and for women and men separately.

Symptoms	*n*	Total (*n* = 866)	Women (*n* = 559)	Men (*n* = 307)
		%	%	%
Tiredness	378	43.6	45.1	41.0
Lower back pain	368	42.5	43.1	41.4
Neck pain	358	41.3	**47.0**	30.9
Headache	335	38.7	**43.1**	30.6
Shoulder pain	308	35.6	**40.1**	27.4
Sleep problems	303	35.0	**38.3**	29.0
Infection	238	27.5	26.5	29.3
Hand/wrist pain	232	26.8	28.8	23.1
Knee pain	220	25.4	25.9	24.4
Cold hands/feet	202	23.3	**25.9**	18.6
Problems concentrating	199	23.0	24.7	19.9
Ankle/foot pain	197	22.7	23.8	20.8
Upper back pain	196	22.6	24.7	18.9
Dizziness	193	22.3	**26.5**	14.7
Flatulence/bowel gas	189	21.8	**25.0**	16.0
Hip pain	184	21.2	**24.3**	15.6
Anxiety	166	19.2	20.4	16.9
Depression	166	19.2	20.6	16.6
Eczema	157	18.1	17.7	18.9
Hot flushes	152	17.6	**20.6**	12.1
Diarrhoea	147	17.0	17.7	15.6
Heart burn/dyspepsia	146	16.9	**19.0**	13.0
Memory problems	143	16.5	17.4	15.0
Dry eyes	137	15.8	**17.7**	12.4
Breathing difficulties	134	15.5	16.8	13.0
Palpitations	120	13.9	**15.9**	10.1
Tinnitus	114	13.2	10.4	**18.2**
Leg cramps	111	12.8	13.6	11.4
Elbow pain	98	11.3	11.8	10.4
Chest pain	92	10.6	8.8	**14.0**
Vomiting	87	10.0	**12.0**	6.5
Fasciculation/twitches	82	9.5	8.1	12.1
Allergy	79	9.1	**10.9**	5.9
Oedema	79	9.1	**10.6**	6.5
Urinary problems	70	8.1	6.8	10.4
Constipation	69	8.0	**10.6**	3.3
Sight problems	47	5.4	6.1	4.2
Fainting	16	1.8	1.3	2.9

*Note*. The symptoms are listed in descending order of overall prevalence. Bold values indicate a significantly higher prevalence of a symptom (*p* < 0.05) in one sex. *p-*Values were calculated using the Chi-squared test.

### Independent variables

From the GP questionnaire, we used only information about the diagnosis and possible chronic conditions. The GPs were asked to register the main diagnosis (only one) by using codes from the second edition of the ICPC (ICPC-2) or by written text, the latter of which two authors (MK and HTM) had labelled with ICPC-2 codes before the analyses. The total number of reported ICPC-2 codes was 321. If more than one main diagnosis was given, which was the case in five of the questionnaires, the first diagnosis written was used. We selected the most frequent single diagnoses for further analyses: asthenia (ICPC A04), diabetes (T89, T90), depression/anxiety (P01, P03, P74, P76), hypertension (K85, K86) and lower back pain (L02, L03, L84, L85).

The GPs were also asked to report whether or not the patient had, in addition to the current diagnosis, one or more prevalent chronic conditions from the following nine diagnostic categories: cardiovascular, respiratory, cancer, musculoskeletal, endocrinological, gastroenterological, psychological, neurological, and other. A sum score of 0, 1, 2, and 3+ prevalent chronic conditions was created. The GP questionnaire contained no questions about the symptoms reported by the patient.

In addition to the symptom checklist, the patient questionnaire included questions about the patients’ sex, age, civil status, educational level, and employment status. Age was pooled into the age categories 18–29, 30–39, 40–49, 50–59, 60–69, and 70+ years. Civil status was grouped into married, separated, widowed, and single. Educational level was registered as ≤10 years, 11–13 years, university (1–4 years), and university (>4 years). Employment status was categorized as employed, social security grant for ≤1 year, social security grant for >1 year, and retired.

### Statistical methods

Frequencies and percentages were used to describe the prevalence of symptoms. The numbers of symptoms were summarized using means, and differences in means between two categories of a nominal variable were identified using the independent *t*-test. One-way analysis of variance (ANOVA) with a Tukey post hoc test was used to compare the mean number of symptoms of a nominal variable with ≥3 categories.

We selected the five most prevalent diagnoses and clustered the rest into ‘other’, which we used as the reference category.

We also modelled the number of symptoms using a Poisson regression model and obtained estimates (incidence rate ratios; IRRs) of the association between each of several possible socio-demographic, diagnosis and condition-count predictors, adjusted for all other predictors in the model. Poisson regression is a convenient model for estimating the association between the number of symptoms reported and various factors like age or diagnosis; the association is expressed as incidence rate ratios (IRR), which represents the change in the number of symptoms in one group relative to the change in the reference group. We fitted three separate Poisson regression models to our data and selected the best model by using the Bayesian information criterion (BIC), which states that the model with the smallest BIC should be selected.

While it is a common practice to model associations with a dichotomous outcome via a binary logistic regression model, the argument for interpreting the odds ratios (ORs) as relative risks (RRs) holds true only in cases where the outcome is rare (prevalence is ≤10%). Since the prevalence of common symptoms in studies is usually high (>10%) and with 38 symptoms to investigate, the RR, which we used as a descriptive statistic rather than an inferential statistic was preferred above OR to describe associations with our dichotomous outcome. The RR was estimated by dividing the probability of having a symptom given a diagnosis by the probability of having the same symptom if the diagnosis is not given (referent). Because the RR is a ratio of two probabilities, it follows that (1) assumptions regarding probability estimation in each group holds and (2) that the probability of having a symptom given a diagnosis in the reference group is >0. RR estimates and their 95% confidence intervals (CIs) are presented in a forest plot. Only the symptoms with significant CIs for RR are presented.

IBM SPSS Statistics 26 and Stata/SE 16 were used to analyse the data. The significance level was set at *α* = 0.05.

## Results

In total, 1024 questionnaire pairs were distributed; 909 patient questionnaires were returned, and 866 had a corresponding answer from the doctor, giving an overall response rate of 84.6%. The mean age was 48.3 years (women, 47.2; men, 50.2 years), and 64.5% of responders were women.

The most frequent symptoms reported during the past week were tiredness (43.6%), lower back pain (42.5%), neck pain (41.3%), headache (38.7%), shoulder pain (35.6%), and sleep problems (35.0%). Women had a significantly higher prevalence than men of 16 of 38 symptoms (*p* < 0.05). Only chest pain and tinnitus (*p* < 0.05) were more commonly reported by men (Table 1).

The number of symptoms reported by each patient ranged from 0 to 32 (out of 38 possible). At least one symptom was reported by 97%, >10 symptoms were reported by 29.2%, and >15 symptoms were reported by about 1%. The overall mean number of symptoms was 7.5 (men, 6.5; women, 8.1) (*p* < 0.01). The highest mean number of symptoms (11.4) was found in patients receiving a social security grant >1 year. Patients aged 40–49 and 50–59 years reported more symptoms than those younger or older, and those with a chronic condition reported more symptoms than those without a chronic condition. Among the selected diagnoses, patients with hypertension reported fewer symptoms (5.6), whereas those with asthenia (11.1) and depression/anxiety (10.7) reported significantly more symptoms than did patients with all other diagnoses (Table 2).

**Table 2.** Mean distribution of the number of symptoms reported in the past 7 days by patients consulting their GP.

Factors	*n*	Mean (95% CI)	*p-*Values*
Sex (ref.: Men)			
Men	307	6.5 (5.9, 7.1)	
Women	559	8.1 (7.6, 8.6)	<0.01
Age group (ref.: 18–29)			
18–29	153	6.7 (6.0, 7.5)	
30–39	165	7.1 (6.3, 7.9)	0.61
40–49	160	8.4 (7.4, 9.3)	0.01
50–59	142	8.8 (7.7, 9.9)	<0.01
60–69	128	6.9 (6.0, 7.8)	0.77
70+	118	7.2 (6.2, 8.1)	0.54
Civil status (ref.: Married)			
Married	575	7.0 (6.6, 7.4)	
Separated	90	9.3 (8.0, 10.6)	<0.01
Widow(er)	45	8.3 (6.7, 9.9)	0.14
Single	156	8.2 (7.3, 9.2)	0.02
Educational level in years (ref.: ≤10)			
≤10	149	7.8 (6.9, 8.7)	
11–13	323	7.9 (7.3, 8.5)	0.87
University (1–4)	240	7.3 (6.6, 8.0)	0.41
University (>4)	154	6.8 (5.9, 7.7)	0.11
Employment status (ref.: Employed)			
Employed	456	6.1 (5.7, 6.6)	
Social security grants (<1 year)	121	9.5 (8.5, 10.5)	<0.01
Social security grants (>1 year)	133	11.4 (10.3, 12.6)	<0.01
Retired	156	6.7 (5.9, 7.5)	0.25
Selected diagnoses (ref.: Other)			
Asthenia	34	11.2 (9.1, 13.2)	<0.01
Diabetes	27	6.3 (3.8, 8.9)	0.34
Depression/anxiety	37	10.7 (8.7, 12.7)	<0.01
Hypertension	56	5.6 (4.5, 6.7)	0.02
Lower back pain	23	8.0 (5.7, 10.3)	0.58
Other	689	7.4 (6.9, 7.8)	
Prevalent chronic conditions (ref.: None)			
None	306	6.2 (5.7, 6.8)	
1	307	7.5 (6.9, 8.2)	0.03
2	163	8.6 (7.6, 9.5)	<0.01
3+	90	10.1 (8.9, 11.3)	<0.01

*ANOVA and a post hoc test were used to identify differences between the categories within the variables.

In the adjusted Poisson regression model, women reported 21% more symptoms than men (Table 3). The age groups 40–49 years and 50–59 years had 17% and 19% more symptoms than the youngest age group. Patients who had received a social security grant for >1 year had 59% more symptoms than those who were employed. Having three or more prevalent chronic conditions was associated with 36% more symptoms compared with those having no chronic condition. Patients with asthenia and depression/anxiety diagnoses reported 44% and 23% more symptoms, respectively, compared with patients with ‘all other’ diagnoses, whereas patients with hypertension reported 26% fewer symptoms. Patients with lower back pain and diabetes did not differ significantly from those with ‘all other’ diagnoses when comparing the number of symptoms.

**Table 3.** Estimates of incidence rate ratios (IRRs) and their 95% confidence intervals (CIs) obtained from the Poisson regression model showing socio-demographic factors and diagnoses given by GPs that were significantly associated with the number of symptoms reported in the past 7 days.

Factors	Unadjusted		Adjusted	
	IRR (95% CI)	*p-*Values	IRR (95% CI)	*p-*Values
Sex (ref.: Men)				
Women	1.24 (1.18, 1.31)	<0.01	1.21 (1.15, 1.28)	<0.01
Age groups (ref.: 18–29)				
30–39	1.05 (0.96, 1.14)	0.28	1.04 (0.95, 1.13)	0.42
40–49	1.24 (1.14, 1.35)	<0.01	1.17 (1.08, 1.27)	<0.01
50–59	1.30 (1.20, 1.42)	<0.01	1.19 (1.09, 1.30)	<0.01
60–69	1.03 (0.94, 1.12)	0.54	0.96 (0.86, 1.06)	0.38
70+	1.06 (0.97, 1.16)	0.19	1.10 (0.95, 1.27)	0.21
Employment status (ref.: Employed)				
Social grants <1 year	1.55 (1.45, 1.66)	<0.01	1.44 (1.34, 1.55)	<0.01
Social grants >1 year	1.87 (1.75, 1.99)	<0.01	1.59 (1.48, 1.71)	<0.01
Retired	1.09 (1.02, 1.17)	0.02	1.04 (0.91, 1.18)	0.58
Selected diagnoses (ref.: All other)				
Asthenia	1.52 (1.37, 1.68)	<0.01	1.44 (1.29, 1.60)	<0.01
Diabetes	0.86 (0.74, 1.00)	0.05	0.97 (0.83, 1.14)	0.72
Depression/anxiety	1.46 (1.32, 1.62)	<0.01	1.23 (1.10, 1.36)	<0.01
Hypertension	0.76 (0.68, 0.85)	<0.01	0.74 (0.66, 0.84)	<0.01
Lower back pain	1.09 (0.94, 1.26)	0.26	1.15 (0.99, 1.33)	0.07
Prevalent chronic conditions (ref.: 0)				
1	1.02 (0.94, 1.11)	0.66	0.99 (0.91, 1.08)	0.83
2	1.15 (1.06, 1.26)	<0.01	1.09 (0.99, 1.19)	0.07
3+	1.46 (1.33, 1.59)	<0.01	1.36 (1.23, 1.51)	<0.01

*Note*. Three separate adjusted Poisson models were fitted to the data for the numbers of symptoms using the Poisson regression models: Model 1: (Bayesian information criterion (BIC) 6028) sex, age, civil status, educational level, employment status, selected diagnoses, chronic conditions (not shown); Model 2: (BIC 6020) sex, age, educational level, employment status, selected diagnoses, chronic conditions (not shown); and Model 3: (BIC 6004) sex, age, employment status, selected diagnoses, chronic conditions. Model 3 (Table 4) was chosen because it has the smallest BIC.

**Figure 1.**
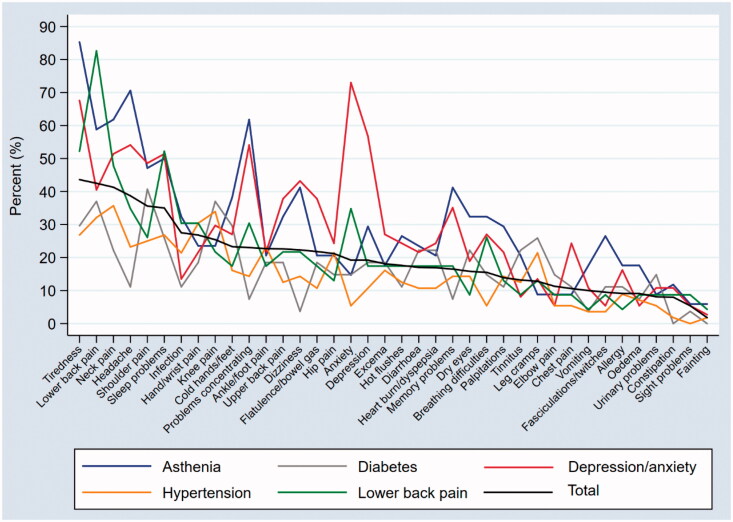
Prevalence of individual symptoms according to the selected diagnoses compared with the total prevalence of symptoms in the study population.

Figure 1 shows the prevalence of the individual symptoms for the five selected diagnoses compared with the total prevalence of symptoms in the study population. The symptoms are presented in descending order of total prevalence. The prevalence rates of the diagnoses of lower back pain, depression/anxiety, and asthenia showed symptom patterns with the same prevalence of symptoms as the total. Hypertension and diabetes followed the total prevalence of the symptoms closely, except for four and two of the 38 symptoms, respectively.

The RR of having each of the symptoms given one of the diagnoses compared with the RR of having the symptoms in those not having the diagnosis is shown in a forest plot (Table 4). In this table, only symptoms with an RR significantly different from 1 are presented.

**Table 4.** Relative risk (RR) estimates showing the likelihood of patients reporting the individual symptoms in the past 7 days according to the most prevalent diagnoses given by their GP.

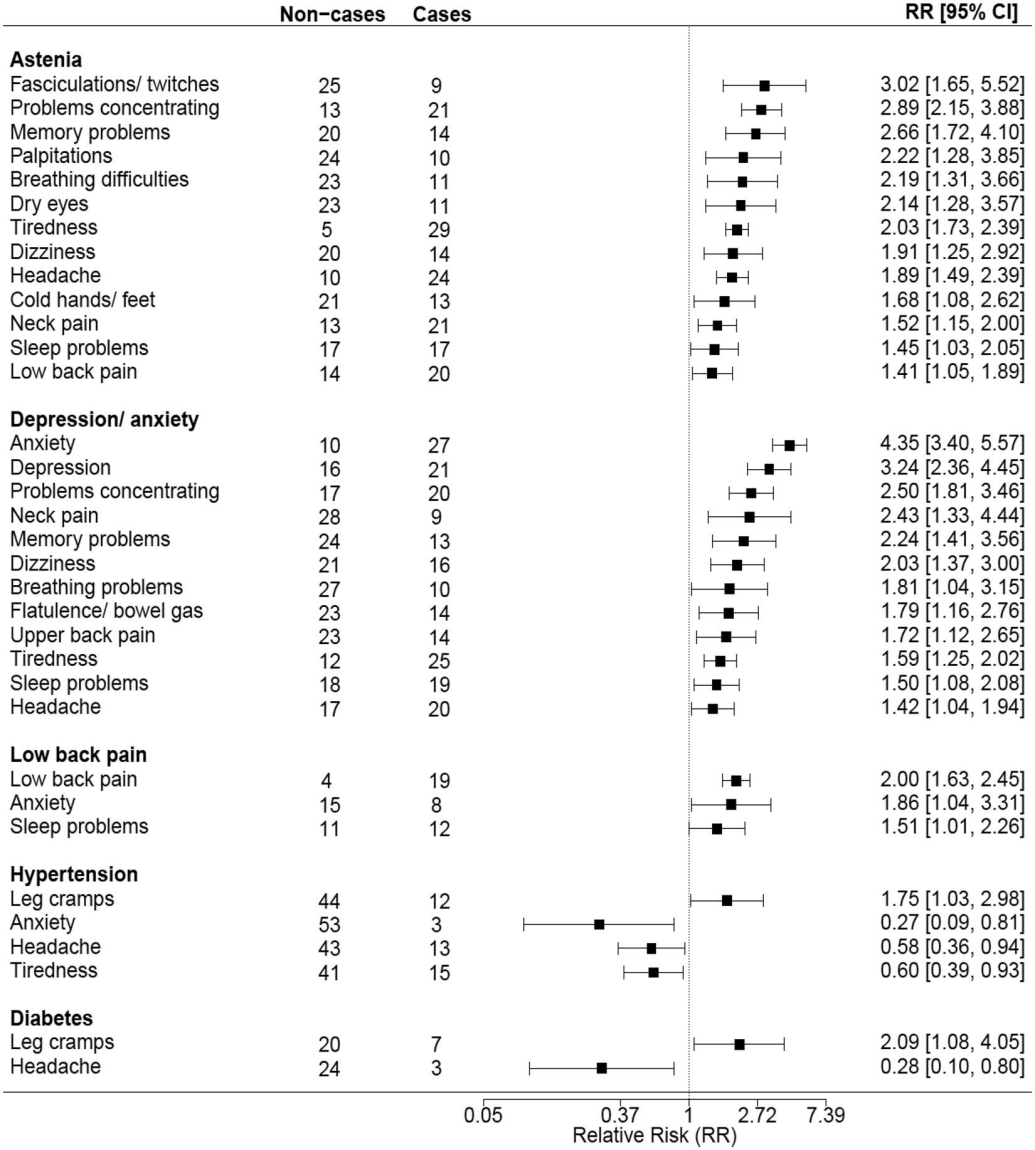

*Note*. Non-cases and cases refers to patients’ not reporting and reporting symptoms, respectively. RRs and their 95% confidence intervals (CIs) to the right of the vertical line (dotted) in the forest plot represent an increase in the likelihood of having a particular symptom, whereas RRs to the left of the vertical line represent a decrease in the likelihood. Only the symptoms with statistically significant CIs are shown in the plot.

Of the 38 symptoms, increased RRs were found for 13 symptoms in patients diagnosed with asthenia, 12 symptoms in those with depression/anxiety, three symptoms in those with lower back pain, one symptom in those with diabetes, and one symptom in those with hypertension. For the rest of the symptoms, no increased RR was found in patients with a diagnosis compared with those without the diagnosis.

We found only small variations in symptom patterns according to age and sex (data not shown).

## Discussion

### Summary of the main findings

Patients in general practice reported a mean of 7.5 symptoms during the week before the consultation. Tiredness, lower back pain, neck pain, headache, shoulder pain, and sleep problems were the most prevalent symptoms. Patients receiving a social security grant and being diagnosed with asthenia or depression/anxiety reported the most symptoms. The prevalence of the symptoms showed similar patterns across the most prevalent diagnoses.

### Strengths and weaknesses of the study

The survey was conducted among consecutive patients seen in general practice. The group of patients is representative of Norwegian adults in terms of age and sex distribution [23], except for slightly more respondents aged 30–49 and slightly fewer older than 70 years. The response rate was high, and the number of participating doctors was acceptable.

The participating GPs were recruited from counselling groups that were part of a postgraduate education required to become a specialist GP. Therefore, the doctors had limited experience. As is the case for most similar studies, the willingness to participate could have led to a selection of GPs with a particular interest in research on symptoms. Awareness of the study may have influenced the GPs in their diagnostic attribution. As we did not focus on outcome measures in meetings with the groups, this potential bias should be minor. The GPs were asked to include consecutive patients on a day in practice, but we do not know whether or how often the GPs forgot to hand out a questionnaire.

The GPs were asked to record only the main diagnosis in the consultation but, for five patients, more than one diagnosis was registered. In retrospect, we should have provided an opportunity to register more than one diagnosis.

Although there has been an increased focus on research on symptoms in recent years, the studies conducted vary in both type and number of symptoms included, and in the inclusion period, which makes comparisons difficult [20,24]. There is no common questionnaire that could facilitate comparisons. In this study, we created the patient questionnaire by merging two commonly used questionnaires.

We did not consider the intensity of symptoms, but previous research has shown that even symptoms considered to be less bothersome are important to self-reported health and functional ability [18].

Our main objective was to map the occurrence of symptoms among patients and to compare these with a number of factors, including the diagnoses given by the doctor. We did not record which symptoms were presented to the GP during the consultation.

An important limitation of our study was the low prevalence of each diagnosis. We, therefore, selected the five most frequent diagnoses for further analysis.

### Findings in relation to other studies

Studies both among patients in general practice and the general population have shown that tiredness, musculoskeletal symptoms, and headache are among the most reported symptoms [1,14,25,26], as we found in our study.

The mean number of symptoms reported was 7.5; in our previous population study, we found the mean number of symptoms was 6.0 [27]. This difference may reflect that the population study including fewer symptoms (22 versus 38). However, one would expect a higher number of symptoms to be reported by people who visit a GP than in population surveys.

Our finding that women reported more symptoms than men is consistent with the results from other studies [1,14,25,26], although the sex difference was significant only for 16 of the 38 symptoms in our study. This sex difference was similar to that found in our population study, with 25% more symptoms in women in our study and 31% more in our previous study [27].

In our study, the middle-aged participants reported more symptoms than the younger and older age groups. This result differs from those of some previous studies [26], but is consistent with the results in large population-based studies [2,14].

Patients receiving a social security grant reported the highest number of symptoms. Presenting many symptoms is known to be strongly associated with low functional status and high rates of absence from work [18,19,28]. Hence, experiencing many symptoms may be considered a sign of impaired health.

As expected, the mean number of symptoms increased with an increasing number of chronic conditions [14]. The differences in the number of symptoms among the most prevalent diagnoses are clinically explainable. Patients with the diagnoses asthenia and depression/anxiety report a high number of symptoms. Asthenia (A04) is a symptom diagnosis according to ICPC-2 and may be used for tiredness/asthenia symptoms alone. However, asthenia is also associated with medically unexplained symptoms [25], which in turn are strongly associated with the reporting of multiple symptoms [29]. Mental health problems are also associated with a high number of symptoms [30]. Diabetes can result in complications in several different organs, but diabetes patients in general practice are often in an early stage, have few diagnosis-specific symptoms, and report good health [31]. Hypertension among general practice patients is as much an asymptomatic risk factor as a disease diagnosis, and most patients with hypertension also rate their health as good [32]. In our study, participants with hypertension had fewer symptoms than the overall mean.

There is often a discrepancy between the experienced symptoms and the symptoms that are revealed in a medical consultation [15]. Although we did not collect information about which symptoms were presented to the GP during the consultation, we have reason to believe that several symptoms were not presented because they may have been seen as irrelevant by the patient and not asked about by the doctor [1].

We have explored symptom patterns for the most prevalent diagnoses. We found differences in the patterns, especially for the diagnoses of depression/anxiety and asthenia, where almost one-third of the symptoms had an increased RR for being reported. These findings suggest some important and clinically expected differences between the diagnoses. However, despite the differences, the selected diagnoses seem to share a common pattern of symptoms. For most symptoms, the RRs in patients with a diagnosis did not differ significantly from those not having the diagnosis. The similarities in the symptom patterns across the diagnoses suggest that symptoms are not necessarily a sign of a particular disease.

Patterns of symptoms have previously been explored using factor analyses [22,33]. A recent Danish study by Eliasen et al. found a strong correlation between symptoms within certain categories, such as the musculoskeletal, gastrointestinal, and cardiopulmonary categories [34]. On the other hand, several studies have demonstrated a general factor that involves loadings from all symptoms. The findings of correlations between symptoms across body regions and organ systems [34,35] confirm that symptom patterns constitute a complex picture.

Reporting a high number of symptoms independent of the type or severity [18] may be indicative of a patient’s future health status [36]. Patients experiencing a high number of symptoms with a high symptom concern or with symptoms that affect daily activities consult a GP more often [2], even though they may present only a selection of the symptoms in the consultation. Insight into the whole pattern of symptoms in patients might provide useful information for clinical evaluations by GPs about whether a patient’s symptoms can be linked to a particular disease.

## Conclusion

Our results indicate that most patients report a variety of symptoms, and that these symptoms appear to be partly independent of the diagnoses given by their GP. Information about the total symptom load may provide a better understanding of the patient’s needs. On the other hand, such information might complicate the diagnostic process because symptoms are not necessarily an indication of disease, contrary to what is implied in the existing definition of symptoms.

## Ethical approval

The study was presented to the Ethics Board (The Regional Committee for Medical and Health Related Research Ethics in Western Norway) but was exempted from review. Research on anonymous data not collected by the researchers themselves are exempted.
